# rCsHscB Derived from *Clonorchis sinensis*: A Carcinogenic Liver Fluke Ameliorates LPS-Induced Acute Hepatic Injury by Repression of Inflammation

**DOI:** 10.3390/pathogens11121548

**Published:** 2022-12-15

**Authors:** Bo Zhang, Chunyang Fan, Qi Tan, Yuzhao Zhang, Qing Jiang, Qian Yu, Beibei Zhang, Kuiyang Zheng, Chao Yan

**Affiliations:** 1 Jiangsu Key Laboratory of Immunity and Metabolism, Department of Pathogenic Biology and Immunology, Jiangsu International Laboratory of Immunity and Metabolism, Xuzhou Medical University, Xuzhou 221004, China; 2 National Experimental Demonstration Center for Basic Medicine Education, Department of Clinical Medicine, Xuzhou Medical University, Xuzhou 221004, China; 3 Department of Orthopaedics, The First Affiliated Hospital of Soochow University, Suzhou 215006, China

**Keywords:** rCsHscB, LPS, sepsis-associated liver injury, MAPK, *Clonorchis sinensis*

## Abstract

Sepsis-associated acute liver injury caused by spillovers of bacteria and endotoxins (lipopolysaccharide, LPS) into the liver remains a public health issue due to the lack of specific therapeutic approaches. Previous studies showed that the recombinant protein HscB (rCsHscB) of *Clonorchis sinensis*, a carcinogenic liver fluke, had an anti-inflammatory effect and could alleviate inflammatory diseases such as enteritis; however, whether it can prevent sepsis-associated acute liver injury induced by LPS is still unknown. In our current study, the therapeutic effects and the potential mechanisms of rCsHscB on LPS-induced acute liver injury were investigated both in vivo and in vitro. The data showed that rCsHscB prevented LPS-induced liver damage, as demonstrated by histopathological observation and hepatic damage markers (the activities of serum ALT and AST) in a murine model of sepsis-associated acute liver injury. rCsHscB also significantly reversed the high levels of serum IL-6 and MCP-1 induced by LPS. In addition, rCsHscB attenuated the production of LPS-induced proinflammatory cytokines, including IL-6 and TNF-α, in a macrophage cell line-RAW264.7, through possible mediation by the MAPK signaling pathway in vitro. In conclusion, the present study demonstrates that rCsHscB derived from a fluke *C. sinensis* protects against sepsis-associated acute liver injury induced by LPS, which may be attributed to the inhibition of the MAPK signaling pathway. Our present study provides a potential therapeutic strategy for sepsis-associated acute liver injury.

## 1. Introduction

Sepsis is a life-threatening illness that results from a systemic dysregulated response to severe infection and leads to multi-organ dysfunction syndrome (MODS) [1]. Sepsis leads to acute liver injury and fulminant hepatic failure, characterized by ischemic hepatitis, sepsis-induced cholestasis, and secondary sclerosing cholangitis [2]. The sepsis-associated acute liver injury also involves in the pathogenesis of MODS and is closely related to an unfavorable prognosis [3]. Despite advances in supportive care, there are still significant challenges to treating sepsis-associated acute liver injury in clinical practice, since no specific and effective therapeutic approaches for sepsis-associated liver injury are available [1].

The exact pathogenic mechanism of sepsis-associated acute liver injury remains unclear, but it is generally considered that sepsis-associated acute liver injury is a pathogenic inflammation mediated by immune system disorders due to endotoxins produced by various microbes [4]. This suggests that the disturbed immune responses due to the overwhelming secretion of Type I immune relative cytokines (proinflammatory cytokines) and the relatively insufficient secretion of Type 2 immune response–relative cytokines (anti-inflammatory cytokines such as IL-10, IL-4) contribute to the pathogenesis of acute liver injuries. Thus, rewiring the imbalanced immune responses has been considered an effective strategy for treating acute liver injury [5]. It is well accepted that LPS-induced acute liver injury is a standard model for mechanistic and therapeutic studies of sepsis-associated acute liver injury. Lipopolysaccharide (LPS), a major component of the outer membrane of Gram-negative bacteria, is a known endotoxin that participates in the development and progression of sepsis-associated acute liver injury [6]. LPS binds with Toll-like receptor 4 expressed on monocytes or neutrophils and induces a series of signaling cascades to active nuclear factor kappa B (NF-κB) and mitogen-activated protein kinases (MAPKs) and triggers the production of proinflammatory cytokines such as IL-6, IL-1β, MCP-1 and TNF-α [7,8,9]. These cytokines and inflammatory mediators directly induce a downstream cascade to promote the apoptosis of hepatocytes through Fas-associated protein with death domain (FADD) [10]. In addition, these cytokines can also recruit more infiltrations of activated neutrophils or monocytes, which produce massive inducible nitric oxide synthase (iNOS) and reactive oxygen species (ROS) to accelerate hepatocyte necrosis by the NAD(P)H oxidase (NOX) pathway [11].

*Clonorchis sinensis* is a liver fluke that mainly inhabits the bile ducts of mammals and causes a severe zoonotic parasitic disease, clonorchiasis, characterized by chronic cholangitis, epithelial hyperplasia, peribiliary fibrosis, and liver cirrhosis [12]. *C. sinensis* is one of the Type I biological carcinogens causing cholangiocarcinoma in humans, according to a report from the World Health Organization [13]. Studies have shown that *C. sinensis* have potent immunoregulatory abilities to induce the tolerance of the host to *C. sinensis* infection, as *C. sinensis* potently induces Type 2 immune responses, and proinflammatory cytokines are less prominent at the chronic stage [14]. 

Molecular chaperone, HscB, derived from *C. sinensis* (CsHscB) is a ~35 KD lipid-protein with 283 amino acids consisting of three domains: DnaJ, Co-chaperone HscB (COHscB), and C-terminal oligomerization (CTO). Previous studies showed that rCsHscB has potent immunoregulatory abilities to reverse overwhelming Type 1 immune responses by triggering high levels of IL-10 in macrophages both in vivo and in vitro [15]. The protein can be detected in the oral sucker, genital pore, vitelline gland, ovary, and testis of adult worms and can be recognized by the sera from infected mice or crude protein-immune mice [15]. Furthermore, our previous study also demonstrates that rCsHscB protects from chronic ulcerative colitis in mice as caused by dextran sodium sulfate (DSS) by repression of proinflammatory cytokines such as IL-6 and TNF-α, as well as by rebalancing of CD4/CD8 populations through the MAPK signaling pathway [16]. However, little is known about the effects of rCsHscB on LPS-induced sepsis-associated liver injury. Given the background, the present aims were to investigate the anti-inflammatory effects of rCsHscB from *C. sinensis* on acute liver injury induced by lipopolysaccharide and to explore the possible underlying mechanisms further. The present study showed that rCsHscB alleviated LPS-induced acute liver injury in mice by inhibiting hepatic inflammation in vivo. We also found that rCsHscB depressed the secretion of proinflammatory cytokines induced by LPS in macrophages in vitro by suppression of the MAPK signaling pathway. The data of the present study suggest that rCsHscB has potent anti-inflammatory activity and may provide a novel and promising therapeutic approach for preventing sepsis-associated acute liver injury.

## 2. Materials and Methods

### 2.1. Animals 

Experiments were conducted under the National Guide for the Care and Use of Laboratory Animals and were permitted by the ethics committee of Xuzhou Medical University. The whole laboratory procedure was performed under the permission (License NO. 2016-SK-04) and surveillance of the Animal Care and Use Committee of Xuzhou Medical University. 

Twenty 6~8-week-old BABL/c mice (ten male mice and ten female mice) purchased from the Vital River Laboratory Animals Co., Ltd. (Beijing, China) were used in the study. Mice were caged in a specific pathogen-free (SPF) environment with an auto 12 h light and 12 h dark cycle, and all the mice were allowed access to food and water ad libitum. 

### 2.2. Experiment Protocol and Tissue Collection

Sepsis-associated acute liver injury was induced as described elsewhere, with minor modification [17]. Briefly, all mice were stochastically grouped into the control group, rCsHscB group, LPS group, and (LPS + rCsHscB) group. For LPS and (LPS + rCsHscB) groups, the mice were intraperitoneally (i.p.) injected with LPS (5 mg/kg body weight per mouse); 20 min later, rCsHscB (1.25 mg/kg body weight per mouse) was intraperitoneally (i.p.) injected into mice in the (LPS + rCsHscB) group and rCsHscB group. The mice from a healthy control group and the LPS group were also simultaneously injected with an equal amount of 0.9% saline. Twelve hours after LPS injection, all mice were weighed and sacrificed. The blood samples were collected from all mice, and sera were isolated by centrifugation at 3000 rpm at room temperature for 30 min and stored at −80 °C for further use. The livers from these mice were quickly removed and dissected for further study. 

### 2.3. Preparation of rCsHscB

rCsHscB was prepared as described elsewhere [18]. Briefly, rCsHscB was routinely generated by *E. coli* (Ec) using pET21a bacterial expression vector containing CsHscB open reading frame (Invitrogen, Carlsbad, CA, USA). Moreover, the recombined protein was purified by nickel affinity followed by diethyl aminoethyl ion-exchange chromatography. The purity of rCsHscB was assessed by SDS-PAGE with coomassie staining or evaluated by western blotting for the histidine-tagged proteins. rCsHscB was further subjected to remove endotoxins using Solution Endotoxin Erasol (Tiandz, Beijing, China). Moreover, the endotoxin concentration in the rCsHscB solution was qualified using Limulus Amebocyte Lysate (LAL, Tiandz, Beijing, China). rCsHscB solution (LPS < 0.1 EU/mL) was used for further study. 

### 2.4. H&E Staining 

To observe histological changes in the liver of mice in a different group, part of the tissue (about 1 cm × 1 cm × 1 cm × 1 cm) was dissected and fixed with 4% formaldehyde solution and paraffin-embedded; then 4 μm thick serial sections were stained with hematoxylin and eosin (H&E). The image of each section was taken, and the infiltrated immune cells were counted using Image-pro Plus software (Media Cybernetics, Rockville, MD, USA).

### 2.5. Determination of Serum ALT and AST Activity

After 12 h of LPS injection, the blood sample from each mouse was collected, and sera were separated. Serum aspartate aminotransferase (ALT) and alanine aminotransferase (AST) were determined to estimate liver injuries according to an automated procedure in the Affiliated Hospital of Xuzhou Medical University.

### 2.6. Cell Culture

A macrophage cell line RAW264.7 was used for in vitro study. The cells with a density of 1 × 10^6^ cells per well in a 24-well cell culture plate were cultured in DMEM containing 10% fetal bovine serum, supplemented with 1% double-antibody, and cultured in a 37 °C, 5% CO_2_ incubator. Macrophages were stimulated by DMEM (negative control), rCsHscB protein (100 ng/mL), LPS (100 ng/mL), and LPS (100 ng/mL) combined with rCsHscB protein (100 ng/mL) for 24 h. The supernatant was collected for subsequent ELISA assays.

### 2.7. ELISA

The levels of MCP-1, IL-10, IL-6, and TNF-α in the sera of mice or culture supernatants were quantified using commercially available ELISA kits (eBioscience, San Diego, CA, USA) according to instructions. 

### 2.8. Western Blot

Cultured macrophages or livers of the mice were collected and lysed by RIPA buffer (Beyotime, Shanghai, China) on ice for 20 min, and liver tissue was homogenized with RIPA buffer on ice, followed by centrifugation at 12,000× *g*, 4 °C for 20 min. The protein concentration was measured by a commercial BCA kit (Beyotime Biotechnology, Shanghai, China). Approximately 30 mg of total protein was separated by 12% SDS-PAGE and then transferred onto polyvinylidene fluoride membranes. The membranes were blocked with 5% skim milk powder at room temperature for 2 h, then overnight at 4 °C with primary antibodies targeting phospho-JNK (Cell Signaling Technology, Danvers, MA, USA; 1:1000 dilution), phospho-ERK (Cell Signaling Technology; 1:1000 dilution), phospho-p38 (Cell Signaling Technology; 1:1000 dilution), and tubulin (Beyotime, Shanghai, China; 1:1000 dilution) as a loading control. Then, the membranes were washed with washing buffer three times and incubated with HRP-conjugated goat anti-rabbit or mouse IgG secondary antibody (proteintech, Wuhan, China; 1:10,000 dilution) for 2 h at room temperature and washed with washing buffer three times. Detection was performed using an enhanced chemiluminescence reagent (Biosharp, Hefei, China). Images were acquired using a Bio-Rad chemiluminescence imaging system and were analyzed with Image J software.

### 2.9. Statistical Analysis

All data are shown as mean ± standard error (SE). The data were analyzed using SPSS 20.0 software (SPSS Inc, Chicago, IL, USA). In our study, to compare the means of more than two different groups, one-way ANOVA followed by the Student-Newman-Keuls (SNK) test was employed; a *p* value of less than 0.05 was deemed statistical difference. 

## 3. Results

### 3.1. rCsHscB Protects against Pathological Changes in Mice with LPS-Induced Liver Injury 

**Figure 1:**
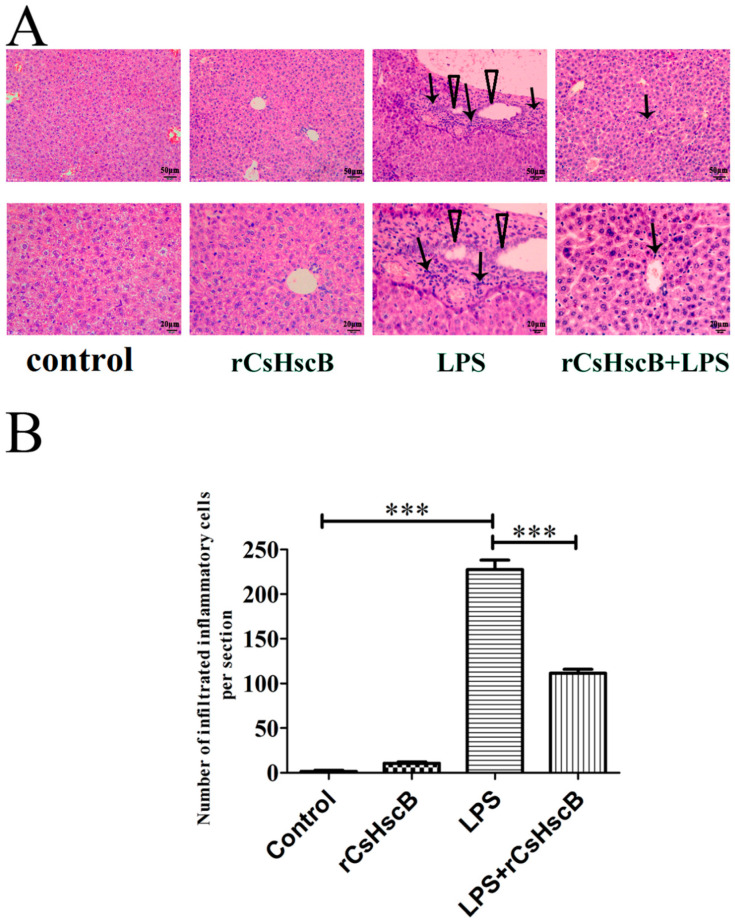
**Histologic changes of liver tissues upon rCsHscB treatment in LPS-induced acute liver injury (H&E staining).** Mice were divided into four groups, including the control group (PBS), rCsHscB (1.25 mg/kg body weight/mouse, i.p.) group, LPS (5 mg/kg body weight per mouse, i.p.) group, and LPS (5 mg/kg body weight per mouse, i.p.) + rCsHscB (1.25 mg/kg body weight per mouse, i.p.) group. For the LPS + rCsHscB group, mice were treated by intraperitoneal instillation of LPS (5 mg/kg body weight per mouse) 20 min before rCsHscB (intraperitoneal injection, 1.25 mg/kg body weight per mouse) injection. Then, 12 h later, mice were anesthetized, and the liver from each mouse was collected for H&E staining. (**A**) Histologic changes of the liver in different groups of mice. Arrow represents the inflammatory cell infiltrations in the liver of mice; the triangle indicates the proliferated biliary epithelium cells. (**B**) Numbers of infiltrated inflammatory cells in the liver of these groups. *** indicates that *p* value is less than <0.001, compared with indicated group.

To evaluate the effect of rCsHscB treatment on sepsis-associated acute liver injury induced by LPS, H&E staining was employed to examine pathological changes of the liver. Compared with the control group, the liver sections from the mice of LPS group showed significant liver injuries, such as cell swelling, focal necrosis, and inflammatory cell infiltration, which suggested that the mouse model for sepsis-associated acute liver injury was established successfully. However, the histopathological lesions of the LPS + rCsHscB group were considerably alleviated after rCsHscB treatment (Figure 1). Furthermore, the infiltrated inflammatory cells around portal areas were significantly decreased after rCsHscB treatment in LPS + rCsHscB group, compared with those in the LPS group (Figure 1A,B, *p* < 0.001). These results indicated that rCsHscB might attenuate the histologic damages in LPS-induced liver injury.

### 3.2. rCsHscB Improves the Liver Damage Caused by LPS

**Figure 2:**
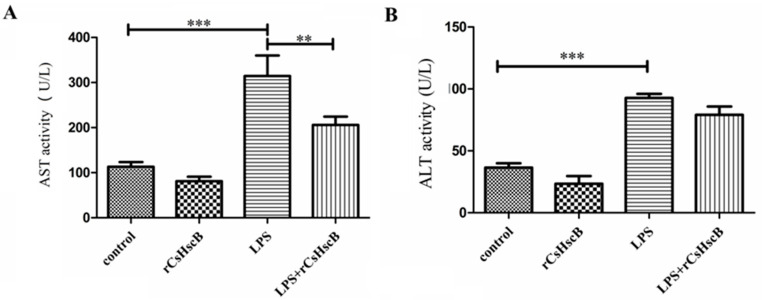
**The effects of rCsHscB on hepatic damage markers ALT and AST in the liver of mice stimulated by LPS.** Mice were divided into four groups: the control group, rCsHscB group, LPS group, and LPS + rCsHscB group. The serum of each mouse from the respective group was collected 12 h after LPS treatment. (**A**) The activity of AST in the sera of mice from each group. (**B**) The activity of ALT in the sera of mice from each group. ** indicates that *p* value was less than 0.01; *** indicates that *p* value is less than <0.001, compared with indicated group.

Serum AST and ALT were determined at 12 h after LPS treatment with or without rCsHscB pretreatment in mice. The levels of serum AST and ALT were both significantly increased in LPS-treated mice compared with the control group (Figure 2A,B, *p* < 0.001). However, rCsHscB treatment restrained the levels of AST triggered by LPS, and there was a statically significant difference between the LPS-stimulated group and rCsHscB treatment group (LPS+ rCsHscB group, *p* < 0.01, Figure 2A). For the serum ALT, the level of ALT was slightly decreased by rCsHscB treatment after LPS-stimulated mice, and this was not statistically different from LPS-stimulated mice (Figure 2B). 

### 3.3. rCsHscB Significantly Reverses LPS-Induced High Levels of IL-6 and MCP-1 in the Sera of Mice

**Figure 3:**
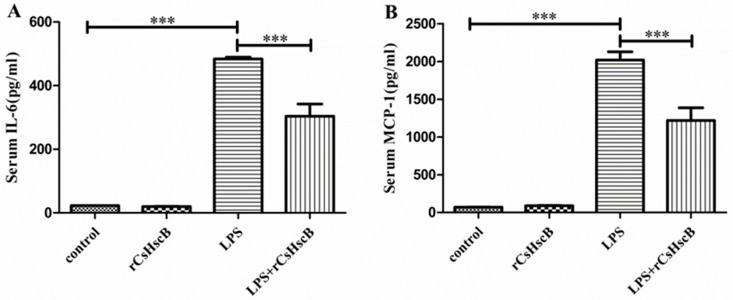
**rCsHscB reduces the secretion of serum IL-6 and MCP-1 in sepsis-associated acute liver injury mice induced by LPS.** Mice were grouped, including the control group, rCsHscB group, LPS group, and LPS + rCsHscB group. The sera of the mice were collected after LPS treatment for 12 h, and the concentrations of IL-6 (**A**) and MCP-1 (**B**) in the sera were determined by ELISA. *** indicates that *p* value is less than <0.001, compared with indicated group.

To assess the treatment of rCsHscB on immune response in LPS-induced sepsis-associated liver injury, the concentrations of proinflammatory cytokines IL-6 and MCP-1 in the sera of mice from each group were detected by commercial kits. As shown in Figure 3, the levels of IL-6 and MCP-1 in sera from control and rCsHscB mice were very low, and there was no difference between these two groups, suggesting that rCsHscB i.p. with 1.25 mg/kg body weight per mouse for 12 h could not induce significant production of IL-6 and MCP-1 in mice. Furthermore, LPS stimulation significantly increased the levels of IL-6 and MCP-1 in the sera from the LPS group, compared with the control group (Figure 3, *p* < 0.001). However, the levels of IL-6 and MCP-1 in the sera from rCsHscB treated mice following LPS stimulation were markedly decreased compared with those from LPS-stimulated mice (Figure 3, *p* < 0.001). 

### 3.4. rCsHscB Suppresses LPS-Induced Macrophage Inflammatory Response

**Figure 4:**
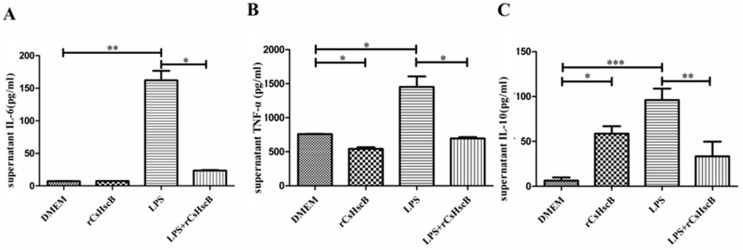
**rCsHscB attenuates LPS-induced inflammatory response in RAW264.7 cells.** RAW 264.7 cells were stimulated with medium (negative control), rCsHscB (100 ng/mL), LPS (100 ng/mL), and LPS (100 ng/mL) + rCsHscB (100 ng/mL) for 24 h, the supernatants were collected, and IL-6 (**A**), TNF-α (**B**) and IL-10 (**C**) in the supernatants of cultured cells were determined by ELISA. * indicates that *p* value was less than 0.05; ** indicates that *p* value was less than 0.01; *** indicates that *p* value is less than <0.001, compared with indicated group.

In the present study, the anti-inflammatory or proinflammatory properties of rCsHscB were assessed using a macrophage cell line RAW264.7. The concentrations of IL-6, TNF-α, and IL-10 in the supernatant of RAW264.7 stimulated with medium, rCsHscB, LPS, or LPS + rCsHscB were evaluated. For IL-6 and TNF-α production, rCsHscB alone could not induce an increased level, compared with a medium group (Figure 4A,B, *p* > 0.05), suggesting that rCsHscB might have no proinflammatory effects, and the effects of endotoxin contamination during the preparation of rCsHscB were limited. However, the concentration of IL-6 and TNF-α were significantly increased in supernatants of LPS-stimulated cells, whereas treatment with rCsHscB significantly decreased the levels of IL-6 and TNF-α that were induced by LPS (decreased about seven times for IL-6 and two times for TNF-α, as shown in Figure 4A,B, *p* < 0.05). For IL-10 production, as shown in Figure 4C, rCsHscB alone could potently induce macrophage secreting high levels of IL-10 (almost five times higher than DMEM control, *p* < 0.001). Similar to IL-6 and TNF-α, rCsHscB also decreased the IL-10 production triggered by LPS (Figure 4C, *p* < 0.01). 

### 3.5. rCsHscB Protects against LPS-Induced Liver Injury by Anti-Inflammation Associated with MAPK Signaling Pathway

**Figure 5:**
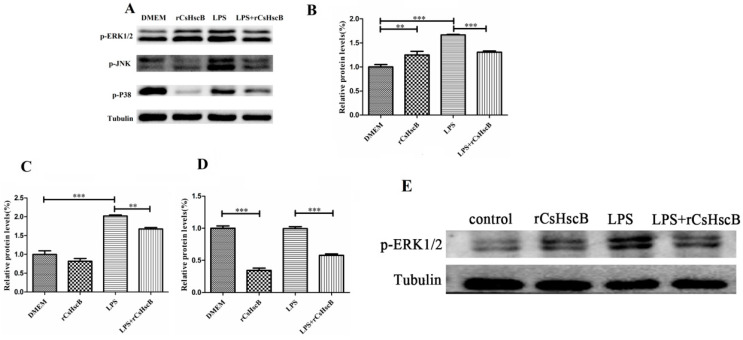
**MAPK pathway may mediate the depression of rCsHscB in LPS-induced acute liver injury.** (**A**–**D**) RAW 264.7 cells were stimulated with medium (negative control), rCsHscB (100 ng/mL), LPS (100 ng/mL), and LPS (100 ng/mL) + rCsHscB (100 ng/mL) for 24 h, the cells were collected and lysed, and phosphorylated proteins of MAPK including ERK, JNK, and p38 in RAW264.7 cells were detected by Western blot (**A**). (**B**) Analysis of phosphorylation of ERK by ImageJ software. (**C**) Analysis of phosphorylation of JNK by Image J. (**D**) Analysis of phosphorylation of p38 by Image J software. (**E**) Part of the liver of mice from the control group, rCsHscB group, LPS group, and LPS + rCsHscB group was lysed, and the phosphorylated ERK1/2 was detected by western blot. ** indicates that *p* value is less than 0.01; *** indicates that *p* value is less than <0.001, compared with indicated group.

To further investigate the potential mechanisms of rCsHscB regarding the anti-inflammatory effects on protection against LPS-induced sepsis-associated liver injury, we tested the MAPK signaling pathway, including ERK1/2, JNK, and P38 since the NF-κB signaling pathway was not involved in rCsHscB-induced cytokine production as demonstrated by our previous study [18]. In vitro, it was found that phosphorylation of ERK1/2 levels (Figure 5A,B, *p* < 0.01), but not p-JNK, was significantly enhanced in the rCsHscB-stimulated group, compared with DMEM group (Figure 5A,C, *p* < 0.01). However, in contrast with the DMEM group, rCsHscB depressed the activation of phosphorylated P38 (Figure 5A,D, *p* < 0.001), suggesting rCsHscB could activate ERK1/2 and depress the phosphorylation levels of P38 directly. Furthermore, LPS stimulation potently increased phosphorylation levels of ERK1/2 and JNK (Figure 5A–C, *p* < 0.001), but the phosphorylation levels of P38 were unchanged in the LPS group (Figure 5D, *p* > 0.05), indicating that LPS may induce the activation of RAW264.7 cells by ERK1/2 and JNK pathways but not the P38 signaling pathway. Meanwhile, compared with the LPS group, rCsHscB suppressed the phosphorylation levels of ERK1/2, JNK, and P38 in macrophages stimulated by LPS combined with rCsHscB (LPS + rCsHscB group) (Figure 5A–D, *p* < 0.01).

In vivo, the phosphorylation levels of ERK1/2 in the liver of the mice following LPS or rCsHscB injection (i.p.) were increased compared with the healthy control (PBS, i.p.) group. However, rCsHscB could depress the phosphorylation levels of ERK1/2 in the liver of LPS-induced sepsis-associated liver injury at 12 h after LPS injection (Figure 5E). Taken together, the data indicate that the rCsHscB exerts its anti-proinflammatory effects by inhibition of the LPS-mediated MAPK pathway.

## 4. Discussion

As early as 1989, Strachan et al. proposed the “hygiene hypothesis” theory, which states that pathogens, including worms, exposed in childhood can help regulate the body’s immune system, establish immune tolerance, and reduce the incidence of autoimmune diseases such as sepsis [19,20]. The “hygiene hypothesis” theory provides a theoretical basis for treating sepsis by helminth infection and its practical antigenic components. From this perspective, there is increasing evidences for potential novel targets derived from helminth can be exploited therapeutically due to its full capacities of immunomodulation [21,22]. For example, ES-62 secreted by a filarial nematode *Acanthocheilonema vitae* and its synthetic small molecule analogs have been demonstrated as a therapeutic potential to protect against arthritis [23,24], asthma [25], and atherosclerosis [26]. Recently, Recombinant Sj16 from *Schistosoma japonicum*, with anti-inflammatory effects, has highlighted potential new therapeutic targets for inflammatory bowel diseases [27]. In the present study, we found that rCsHscB derived from a liver fluke could attenuate LPS-induced sepsis-associated acute liver injuries by inhibiting hepatic inflammation both in vivo and in vitro, which may provide a novel therapeutic strategy for sepsis-associated acute liver injury.

The importance of the induction of inflammatory cytokines in LPS-mediated liver injury has been highlighted in many liver disease models [6]. Previous studies have shown that the proinflammatory cytokines such as IL-6 and MCP-1 as the primary mediators accelerated liver damage in LPS-induced acute liver failure in the early stage [28]. LPS, as the main component of the outer membrane of Gram-negative bacteria, potently induced the release of proinflammatory cytokines such as TNF-α, IL-6, and MCP-1 by various immune cells such as macrophage, DC, and neutrophils [29]. These proinflammatory cytokines can induce the apoptosis of hepatocytes and cholangiocytes, which further accumulate the recruitment of these cells to cause severe damage to the liver [30]. In the present study, we found that the systemic inhibition of TNF-α, IL-6, and MCP-1 production by rCsHscB in the mice was parallel to the reduction of hepatic inflammatory cells. Furthermore, in our present study, we also found that the activities of liver enzymes (AST and ALT), which indicated hepatic dysfunction and damaged the structural integrity of hepatocytes, decreased after rCsHscB treatment in mice due to LPS administration. However, ALT was slightly decreased in the LPS + rCsHscB group compared with the LPS group.

During sepsis-associated liver injury, macrophages play a central role in liver inflammation and host defense against infection. Kupffer’s cells (resident macrophages in the liver) and bone marrow-derived macrophages were activated and expressed signaling molecules with the secretion of many inflammatory meditators in response to hepatocyte injury caused by various insults [30]. In LPS-induced acute liver injury, binding of LPS to TLR4 could induce a cascade of NF-κB and MAPK (p38, ERK, and JNK) signaling, resulting in subsequent proinflammatory cytokine overexpression [31,32]. However, our present study found that the MAPK, not NF-κB, signaling pathway was activated in macrophages stimulated by rCsHscB, although the mechanisms were not fully understood. Consistent with other similar studies [33,34,35,36,37], we found that rCsHscB reduced the phosphorylation levels of ERK1/2, JNK, and p38 in macrophages induced by LPS, and proinflammatory cytokines such as TNF-α and IL-6 were also decreased, suggesting that rCsHscB attenuates LPS-induced acute liver injury by repression of the MAPK signaling pathway probably.

In conclusion, the data of the present study suggest that rCsHscB derived from a fluke *C. sinensis* protects against sepsis-associated acute liver injury induced by LPS. The therapeutic effect of rCsHscB might be attributed to the inhibition of the MAPK signaling pathway in LPS-induced inflammatory responses, which may offer a new opportunity for curing sepsis.

## Data Availability

The authors confirm that the data supporting the findings of this study are available within the manuscript.
